# High applicability of ASO-RQPCR for detection of minimal residual disease in multiple myeloma by entirely patient-specific primers/probes

**DOI:** 10.1186/s13045-016-0336-4

**Published:** 2016-10-11

**Authors:** Yinlei Bai, Kwan Yeung Wong, Tsz Kin Fung, Chor Sang Chim

**Affiliations:** Department of Medicine, Queen Mary Hospital, The University of Hong Kong, Pokfulam, Hong Kong

**Keywords:** Multiple myeloma, Minimal residual disease, Patient-specific ASO-RQPCR

## Abstract

Allele-specific oligonucleotide real-time quantitative PCR (ASO-RQPCR) is a standardized technique for detection and monitoring of minimal residual disease (MRD) in acute lymphoblastic leukemia (ALL) but not multiple myeloma (MM) due to a low applicability inherent with presence of somatic hypermutation. Herein, by a staged PCR approach and sequencing, clonality and tumor-specific complementarity-determining region 3 (CDR3) sequence were identified in 13/13 MM by sequential PCR of IgH VDJ (*n* = 10), IgH DJ (*n* = 2), or IgK VJ (*n* = 1). Using consensus primers/probes conventionally employed in ALL, ASO-RQPCR worked in three (23.1 %) cases only. Conversely, using entirely patient-specific primers/probes, ASO-RQPCR was applicable in eight (61.5 %) cases with a sensitivity of 5 × 10^−4^–10^−5^. Moreover, using standard curves constructed by serial dilution of plasmids cloned with patient-specific CDR3, ASO-RQPCR was successful in 12 (92.3 %) cases with a sensitivity of 10^−4^–10^−5^, but not in a case lacking an N region, in which design of a tumor-specific ASO primer was precluded. Finally, in a patient in complete response (CR), further reduction of MRD after autologous stem cell transplantation (ASCT) was demonstrated. In summary, using entirely patient-specific primers/probes, ASO-RQPCR was applicable in >90 % MM patients and enabled detection of dynamic changes of MRD before and after ASCT despite conventional CR.

**Electronic supplementary material:**

The online version of this article (doi:10.1186/s13045-016-0336-4) contains supplementary material, which is available to authorized users.

## Letter to the editor

Minimal residual disease (MRD) in multiple myeloma (MM) may be studied by allele-specific oligonucleotide real-time quantitative PCR (ASO-RQPCR), first applied in acute lymphoblastic leukemia (ALL) using patient-specific ASO forward primers derived from complementarity-determining region 3 (CDR3) sequences of immunoglobulin (Ig) genes [1], combined with consensus reverse germline primers and TaqMan probes in the J_H_ region [2]. While applicable in >90 % ALL [3], the consensus primers/probes are only applicable in 42–75 % MM due to somatic hypermutations [4, 5].

Herein, in 13 consecutive MM (Additional file 1: Table S1), clonality and sequence of CDR3 were identified in diagnostic BM of all 13 (100 %) cases by a staged PCR approach, involving sequential PCR of IgH VDJ, IgH DJ, and IgK VJ rearrangements (Additional file 2: Figure S1; Table S2), superior to studies using BIOMED-2 multiplex PCR [4, 5]. Clonality was identified in three (23.1 %) cases by FR3 IgH VDJ PCR, seven (53.8 %) by FR1 IgH VDJ PCR, two (15.4 %) by IgH DJ PCR, and one (7.7 %) by IgK VJ PCR. Similar to previous MM studies [5–7], V_H_1, V_H_3, and V_H_4 families were mostly used (12~19 %, 47~63 %, and 16~20 %, respectively) (Additional file 2: Table S3).

ASO-RQPCR was first performed using ASO forward primers and consensus primers/probes, according to the EuroMRD guidelines (Additional file 2: Figure S2; Table S2) [2, 8, 9]. By standard curves serially diluted from diagnostic BM, ASO-RQPCR was applicable in only three (23.1 %) cases, with a sensitivity of 5 × 10^−4^–10^–4^ and a quantitative range (QR) of 10^−3^–10^−4^ (Table 1), suggestive of MRD quantification in follow-up samples with one myeloma cell in a background of 10^4^ normal cells [10]. Causes of failures in other cases included absence of amplification (*n* = 5) or non-specific amplification in normal control (*n* = 1) during preliminary qualitative PCR, suboptimal QR of 10^−2^ (*n* = 3), or shortage of diagnostic BM for standard curve (*n* = 1).

**Table 1:** Performance of RQPCR by consensus germline or entirely patient-specific primers/probes

Cases	Consensus germline primers/probes				Patient-specific primers/probes							
	Diagnostic marrow DNA as standards				Diagnostic marrow DNA as standards				CDR3-cloned plasmid as standards			
	Sensitivity	QR	*R* ^2^	Slope	Sensitivity	QR	*R* ^2^	Slope	Sensitivity	QR	*R* ^2^	Slope
Dx53	N/A	10^−2^	N/A	N/A	10^−5^	10^−4^	0.996	−3.30	10^−5^	10^−4^	0.992	−3.44
M3	ND	ND	ND	ND	5 × 10^−4^	5 × 10^−4^	0.989	−3.48	10^−4^	10^−4^	0.997	−3.54
M8	10^−4^	5 × 10^−4^	0.985	−3.38	10^−4^	10^−3^	0.999	−3.46	10^−4^	5 × 10^−4^	0.999	−3.61
M4	ND	ND	ND	ND	10^−4^	10^−3^	0.984	−3.60	10^−5^	10^−3^	0.999	−3.54
Dx31	ND	ND	ND	ND	10^−4^	5 × 10^−4^	0.998	−3.45	10^−4^	10^−4^	0.998	−3.47
M5	10^−4^	10^−4^	0.995	−3.41	5 × 10^−4^	5 × 10^−4^	0.995	−3.67	10^−5^	10^−4^	0.998	−3.51
M10	N/A	5 × 10^−4^	N/A	N/A	N/A	5 × 10^−4^	N/A	N/A	10^−4^	10^−4^	0.994	−3.51
Dx49	5 × 10^−4^	10^−3^	0.982	−3.37	10^−4^	10^−3^	0.986	−3.26	10^−4^	5 × 10^−4^	0.999	−3.76
M1	ND	ND	ND	ND	5 × 10^−4^	10^−3^	0.986	−3.48	10^−4^	5 × 10^−4^	0.998	−3.52
M6	ND	ND	ND	ND	N/A	5 × 10^−4^	N/A	N/A	10^−5^	5 × 10^−4^	0.997	−3.72
M2	N/A	N/A	N/A	N/A	ND	ND	ND	ND	ND	ND	ND	ND
M7	N/A	10^−2^	N/A	N/A	N/A	10^−2^	N/A	N/A	10^−4^	10^−3^	0.999	−3.56
M9	N/A	10^−2^	N/A	N/A	N/A	10^−2^	N/A	N/A	10^−4^	10^−4^	0.997	−3.55

*Abbreviations*: *QR* quantitative range, *R* ^*2*^ correlation coefficient, *N/A* not applicable, *ND* not done due to absence of target band in qualitative PCR or no need (for M2 in patient-specific approach)

To account for a low applicability using consensus primers/probes, a systematic search for sequences mismatches against consensus primers/probes was performed. Sequencing data revealed mismatches in five (50 %) cases against consensus primers, six (50 %) against probes, and two (20 %) against both sites, but not in two IgH cases (M7 and M10) or one IgK case (M9) (Table 2). While a previous MM study also showed a high frequency of mismatches against consensus primers/probes in patients with IgH VDJ rearrangements (herein 80 vs. 75 %), the number of mismatches per case was higher in our study (five vs. three) [11]. Given that mismatches of ≥3 bases would preclude a successful ASO-RQPCR [11], the use of patient-specific primers/probes is inevitable in Chinese MM with profound mismatches than Caucasian patients.

**Table 2:** Mismatches against consensus germline reverse primers and/or probes

Cases	Mismatches against primers			Mismatches against probes		
	Primers	Primer binding site sequences	No.	Probes	Probe binding site sequences	No.
Dx53	R-JH6-intron	gcagaGaGGaaGggccctagagt	4	T-JH6	cacggtcaccgtctcctcaggCaagaa	1
M3	R-JH4-intron	cagagAAaaTATCACagagaggttgt	8	T-JH1.2.4.5	ccctggtcaccgtctcctcaggtg	0
M8	R-JH4-intron	cagagCtaaagcaggagagaggttgt	1	T-JH1.2.4.5	ccctggtcaccgtctcctcaggtg	0
M4	R-JH4-intron	cagGAttTaagTaggGgagaCgttgt	6	T-JH1.2.4.5	ccctggtcaccgtctcctcaggtg	0
Dx31	R-JH4-intron	TagaCCCaaagTaggagagaCAttAt	8	T-JH1.2.4.5	Tcctggtcaccgtctcctcaggtg	1
M5	R-JH3-intron	aggcagaaggaaagccatcttac	0	T-JH3	cCagggacaatggtcaccgtctcttca	1
M10	R-JH6-intron	gcagaaaacaaaggccctagagt	0	T-JH6	cacggtcaccgtctcctcaggtaagaa	0
Dx49	R-JH6-intron	–	?	T-JH6	cacggtcaccgtctcctcaggtaagaT	1
M1	R-JH4-intron	–	?	T-JH1.2.4.5	cTGt---	≥2
M6	R-JH3-intron	–	?	T-JH3	caagggacaat---	?
M2	R-JH4-intron	cagagttaaagcaggagagaggttgt	0	T-JH1.2.4.5	ccctggtcGccgtctcctcaggtg	1
M7	R-JH5-intron	agagagggggtggtgaggact	0	T-JH1.2.4.5	ccctggtcaccgtctcctcaggtg	0
M9	R-Jk1	gatcacttcatagacacagggaacag	0	T-Jk1	tggaaatcaaacgtgag	0

Uppercase letters in sequences indicate mismatches. “?” indicates mismatches cannot be identified based on available sequencing data

Therefore, ASO-RQPCR using entirely patient-specific primers/probes was performed (Additional file 2: Figure S2; Table S4). Except one case (M2) amplified by IgH DJ PCR, where the absence of an N region precluded design of a specific ASO primer, patient-specific primers/probes ASO-RQPCR was successful in eight (61.5 %) cases (Table 1).

Moreover, as diagnostic BM was limited, we attempted to construct standard curves by plasmid DNA cloned with patient-specific CDR3, whereby ASO-RQPCR was applicable in all 12 cases, with a sensitivity of ≤10^−4^ in all 12 (100 %) cases and a QR ≤5 × 10^−4^ in ten (83.3 %) cases (Table 1). Collectively, using CDR3-cloned plasmid DNA, more cases achieved a sensitivity of ≤10^−4^ than using diagnostic BM (12 vs. 5 cases: *p* = 0.0046; Table 1), and applicability was increased from 61.5 to 92.3 %. Moreover, plasmid DNA provides an unlimited supply for standard curve construction in multiple MRD analysis. Furthermore, the use of plasmid DNA is more accurate for cases in which the percentage of plasma cells was not specified in the diagnostic BM.

Finally, in a patient (Dx53) who achieved serological complete response (CR) after four cycles of VTD induction, by ASO-RQPCR with a sensitivity of 10^−5^ and QR of 10^−4^, MRD was present in both BM samples on days 120 and 16 prior to autologous stem cell transplantation (ASCT), but turned negative on day 14 after ASCT, testifying further reduction of tumor load below clinical detection limit by ASCT (Additional file 2: Figure S3).

In summary, a high applicability and sensitivity of MRD detection by ASO-RQPCR can be achieved by clonality detection using stepwise PCR approach, mismatch analysis, and hence entirely patient-specific primers/probes in addition to standard curve construction using CDR3-cloned plasmid DNA. Moreover, the use of ASCT in a patient achieving serological CR after induction was justified by further reduction of tumor load illustrated by ASO-RQPCR. Finally, the frequency and intensity of mismatches against consensus primers/probes between different populations warrants further study in an expanded cohort.

## Additional files

Additional file 1: Materials and methods and Table S1 patient demographics. (DOCX 67 kb)
Additional file 2: Figure S1. Schematic diagram. **Figure S2.** Principles of ASO-RQPCR. **Figure S3.** MRD detection. **Table S2.** Clonality and ASO primers. **Table S3.** Family usage. **Table S4.** Patient-specific primers/probes and downstream primers. (DOCX 2290 kb)

## Abbreviations

ALL: Acute lymphoblastic leukemia
ASCT: Autologous stem cell transplantation
ASO-RQPCR: Allele-specific oligonucleotide real-time quantitative PCR
CDR: Complementarity-determining region
CR: Complete response
Ig: Immunoglobulin
MM: Multiple myeloma
MRD: Minimal residual disease
