# Supplementary material for: High applicability of ASO-RQPCR for detection of minimal residual disease in multiple myeloma by entirely patient-specific primers/probes
Source: J Hematol Oncol. 2016 Oct 11;9:107. doi: 10.1186/s13045-016-0336-4 (PMC5057274; doi:10.1186/s13045-016-0336-4)
Supplement: Additional file 1: — Materials and methods and Table S1 patient demographics. (DOCX 67 kb) [file 13045_2016_336_MOESM1_ESM.docx]

***Materials and Methods***

Patient samples

Thirteen consecutive Chinese MM patients received four cycles of induction with bortezomib-thalidomide-dexametahsone (VTD: bortezomib 1.3mg/m^2^ on D1, 8, 15, 22; thalidomide 200mg daily, dexamethasone 40mg/week), followed by ASCT and thalidomide maintenance (thalidomide 50mg daily for 12 months) were enrolled in this study [[1](#_ENREF_1)].

A staged approach with vincristine, adriamycin, and dexamethasone followed by bortezomib, thalidomide, and dexamethasone before autologous haematopoietic stem cell transplantation in the treatment of newly diagnosed MM [[2](#_ENREF_2)]. Treatment response was defined according to the IMWG criteria [[3](#_ENREF_3)]. The study was approved by the Institutional Review Board of the University of Hong Kong/Hospital Authority Hong Kong West Cluster with informed consents. Patient demographics were summarized in Additional file 1 Table S1.

Genomic DNA was extracted from bone marrow buffy coat or ficolled bone marrow using QIAamp DNA Blood Mini Kit (Qiagen, Hilden, Germany), according to the manufacturer’s instructions. Quality of diagnostic marrow DNA was verified by PCR of the ubiquitous human beta-globin gene using KM29/RS42 primers [[4](#_ENREF_4)]. Plasma cell percentage of the diagnostic marrow was revealed by cytologic examination of Wright-Giemsa stained marrow smear.

Identification of clonal Ig gene rearrangements

Clonal Ig gene rearrangement was identified in the diagnostic marrow sample of each patient. Genomic DNA was sequentially amplified by PCR for IgH complete VDJ, incomplete DJ and IgK VJ rearrangements. IgH rearrangements were first amplified by singleplex PCR using a framework 3 (FR3) consensus primer with a consensus joining primer J_H_21 [[5](#_ENREF_5)]. If clonal rearrangement was not found, sequential singleplex PCR using V_H_ framework 1 (FR1) and D_H_ family-specific primers with a J_H_ consensus primer would be performed [[6](#_ENREF_6)]. IgK VJ rearrangements was amplified using three VK family-specific forward primers with two JK consensus reverse primers in multiplex [[6](#_ENREF_6)]. All PCR products were subjected to polyacrylamide gel electrophoresis, followed by heteroduplex analysis [[6](#_ENREF_6)]. Major PCR bands were excised and purified from gel, followed by bidirectional direct sequencing. In samples with clonal Ig rearrangements, the CDR3 sequence would be revealed by sequence analysis using the IMGT V-QUEST tool (http://www.imgt.org) for identification of V-N-D-N-J segments in those with clonal IgH VDJ, and V-N-J in those with clonal IgK VJ rearrangements. Incomplete IgH DJ rearrangements were analyzed through BLAST (accession number EMB/X97051, http://www.ncbi.nlm.nih.gov/blast/) for the identification of D-N-J segments.

Design of ASO primers, consensus germline and patient-specific primers/probes

Primers and probes were designed with Primer Express version 3.0.1 (Life Technologies) and Primer-BLAST (NCBI). ASO forward primers complementary to the CDR3 regions were designed as previously described [[5](#_ENREF_5)] , and summarized in Additional file 2 Table S2. Consensus germline reverse primers/probes described for MRD study in ALL were employed [[5](#_ENREF_5)]. In case of presence of sequence mismatch at the priming or binding regions of consensus germline reverse primers/probes [[5](#_ENREF_5)], patient-specific reverse primers and Taqman probes were designed against the J_H_ intron and J_H_ exon respectively, and hence an entirely patient-specific primers/probes approach. On the other hand, in case that mismatches could not be fully identified, ASO reverse primers were designed instead, and ASO RQ-PCR was performed with VH FR3-derived patient-specific forward primers and Taqman probes. Probes were designed in the same strands with ASO primers. FAM was chosen as 5’ reporter dye and TAMRA as 3’ quencher. Sequences for patient-specific primers/probes are listed in Additional file 2 Table S4.

Germline J_H_ or J_K_ reverse primers/probes, and primers for control albumin gene were previously described [[5](#_ENREF_5), [7](#_ENREF_7), [8](#_ENREF_8)]. Of note, the probe of albumin gene was modified to T-Albumin 5’-ATGCTGAAACATTCACCTTCCATGCAGA-3’, based on previously described [[8](#_ENREF_8)].

Analysis of mismatches against consensus germline primers/probes

Mismatches against the regions of consensus germline J_H_ (or J_K_) reverse primers/probes were identified by PCR using ASO forward primers and J_H_ (or J_K_) family-specific reverse primers, located at the J_H_ (or J_K_) intron downstream to the consensus germline reverse primers (Additional file 2: Table S4), followed by bidirectional direct sequencing. Sequence data were compared with consensus germline J_H_ (or J_K_) germline reverse primers/probes [[5](#_ENREF_5), [7](#_ENREF_7)]. Alternatively, mismatches against the consensus germline probes could also be detected by PCR using ASO forward primers and consensus germline reverse primers, or PCR using IgH FR1 family-specific primers and a J_H_ consensus primer.

Evaluation of ASO primers

The validity of each ASO primer was first evaluated by a qualitative PCR with the corresponding consensus germline or patient-specific primers. Any target amplification present exclusively in the diagnostic but not normal control DNA (pooled from the peripheral blood buffy coat of 5-10 individual donors) was confirmed by sequencing. Moreover, each ASO primer might be further verified by a pilot ASO RQ-PCR, using the diagnostic sample at the dilutions of 10^-3^ and/or 10^-4^ and the normal control DNA as templates. A valid ASO primer was associated with a positive signal in the dilution of 10^-3^ and/or 10^-4^ and absence of amplification signal (i.e. C_T_ > 40) in the normal control DNA.

Standard curves, ASO RQ-PCR and MRD detection

Standard curves were constructed by 10-folded serial dilution of diagnostic marrow DNA or plasmid DNA cloned with the patient-specific Ig CDR3 sequences. Serial dilution of diagnostic marrow DNA into a normal control DNA ranged from 10^-1^ to 10^-5^ (with an additional 5×10^-4^). Similarly, serial dilution of CDR3-cloned plasmid DNA with normal control DNA ranged from 10^6^ copies to 1 copy (with an additional 50 copies). For construction of the standard curve, ASO RQ-PCR of serial dilutions and no-template control were performed in duplicate, whereas normal control DNA in six replicates. For MRD study of the follow-up marrow samples, ASO RQ-PCR was performed in triplicate. Each reaction consisted of 500 ng genomic DNA in a final volume of 25 µl containing 12.5 µl 2× TaqMan^®^ Universal Master Mix II, with UNG (Applied Biosystems), 300 nM forward and reverse primers, and 200 nM Taqman probe. Reactions were loaded into a 96-well optical plate and run in a StepOnePlus Real-Time PCR System (Applied Biosystems) at 50°C for 2 min, 95°C for 10 min, followed by 50 cycles of 95°C for 15s and 60°C (or 64°C for 1 case) for 1 min. Sensitivity and quantitative range were assessed in accordance to the EuroMRD guidelines [[9](#_ENREF_9)].

MRD level of a follow-up marrow sample was determined by the standard curve and the number of input cells normalized by RQ-PCR of albumin gene [[8](#_ENREF_8)], and expressed as a number of Ig copies per 10^5^ cells ([Ladetto*, et al* 2010](#_ENREF_10)). RQ-PCR data was interpreted in accordance to the EuroMRD guidelines [[9](#_ENREF_9)].

Statistics

Fisher's exact test was employed to compare the sensitivity between ASO RQ-PCR standard curves made by serial dilution of diagnostic marrow DNA and plasmid DNA. A two-sided p-value ≤ 0.05 was considered as statistically significant.

***References***

1. Chim CS, Lie AK, Chan EY, Liu HS, Lau C, Yip S, et al. Treatment outcome and prognostic factor analysis in transplant-eligible Chinese myeloma patients receiving bortezomib-based induction regimens including the staged approach, PAD or VTD. Journal of Hematology & Oncology. 2012; 5:28.

2. Chim CS, Lie AKW, Chan EYT, Leung Y, Cheung SCW, Chan SYT, et al. A staged approach with vincristine, adriamycin, and dexamethasone followed by bortezomib, thalidomide, and dexamethasone before autologous hematopoietic stem cell transplantation in the treatment of newly diagnosed multiple myeloma. Annals of Hematology. 2010; 89:1019-27.

3. Rajkumar SV, Harousseau JL, Durie B, Anderson KC, Dimopoulos M, Kyle R, et al. Consensus recommendations for the uniform reporting of clinical trials: report of the International Myeloma Workshop Consensus Panel 1. Blood. 2011; 117:4691-5.

4. Saiki RK, Chang CA, Levenson CH, Warren TC, Boehm CD, Kazazian HH, Jr., et al. Diagnosis of sickle cell anemia and beta-thalassemia with enzymatically amplified DNA and nonradioactive allele-specific oligonucleotide probes. N Engl J Med. 1988; 319:537-41.

5. Verhagen OJ, Willemse MJ, Breunis WB, Wijkhuijs AJ, Jacobs DC, Joosten SA, et al. Application of germline IGH probes in real-time quantitative PCR for the detection of minimal residual disease in acute lymphoblastic leukemia. Leukemia. 2000; 14:1426-35.

6. van der Velden V, Hochhaus A, Cazzaniga G, Szczepanski T, Gabert J, Van Dongen J. Detection of minimal residual disease in hematologic malignancies by real-time quantitative PCR: principles, approaches, and laboratory aspects. Leukemia. 2003; 17:1013-34.

7. van der Velden VH, de Bie M, van Wering ER, van Dongen JJ. Immunoglobulin light chain gene rearrangements in precursor-B-acute lymphoblastic leukemia: characteristics and applicability for the detection of minimal residual disease. Haematologica. 2006; 91:679-82.

8. Pongers-Willemse MJ, Verhagen OJ, Tibbe GJ, Wijkhuijs AJ, de Haas V, Roovers E, et al. Real-time quantitative PCR for the detection of minimal residual disease in acute lymphoblastic leukemia using junctional region specific TaqMan probes. Leukemia. 1998; 12:2006-14.

9. van der Velden VH, Cazzaniga G, Schrauder A, Hancock J, Bader P, Panzer-Grumayer ER, et al. Analysis of minimal residual disease by Ig/TCR gene rearrangements: guidelines for interpretation of real-time quantitative PCR data. Leukemia. 2007; 21:604-11.

**Table S1** Patient demographics

|  | | No. of Patients |  | Percentage (%) |
| --- | --- | --- | --- | --- |
| Total | | 13 |  | 100 |
| Age, years | |  |  |  |
|  | Range | 48-65 |  | |
|  | Median | 59 |  | |
| Sex | |  |  |  |
|  | Male | 8 |  | 62 |
|  | Female | 5 |  | 38 |
| ISS stage | |  |  |  |
|  | Ⅰ | 4 |  | 31 |
|  | Ⅱ | 3 |  | 23 |
|  | Ⅲ | 6 |  | 46 |
| Ig isotype | |  |  |  |
|  | IgA | 3 |  | 23 |
|  | IgD | 1 |  | 8 |
|  | IgG | 6 |  | 46 |
|  | Light chain | 3 |  | 23 |
| Response after induction | |  |  |  |
|  | CR | 2 |  | 15 |
|  | nCR | 3 |  | 23 |
|  | VGPR | 7 |  | 54 |
|  | PR | 1 |  | 8 |
| Response after ASCT | |  |  |  |
|  | CR | 9 |  | 69 |
|  | VGPR | 3 |  | 23 |
|  | PR | 1 |  | 8 |
| BMPC% of diagnostic marrows | | |  |  |
|  | Range | 10%-95% |  |  |
|  | Median | 60% |  |  |

ISS: International Staging System; CR: complete response; nCR: near complete response; VGPR: very good partial response; PR: partial response; BMPC%: percentage of bone marrow plasma cells, revealed by cytologic examination of Wright-Giemsa stained marrow smear.
