# Supplementary material for: High applicability of ASO-RQPCR for detection of minimal residual disease in multiple myeloma by entirely patient-specific primers/probes
Source: J Hematol Oncol. 2016 Oct 11;9:107. doi: 10.1186/s13045-016-0336-4 (PMC5057274; doi:10.1186/s13045-016-0336-4)
Supplement: Additional file 2: Figure S1. — Schematic diagram. Figure S2. Principles of ASO-RQPCR. Figure S3. MRD detection. Table S2. Clonality and ASO primers. Table S3. Family usage. Table S4. Patient-specific primers/probes and downstream primers. (DOCX 2290 kb) [file 13045_2016_336_MOESM2_ESM.docx]

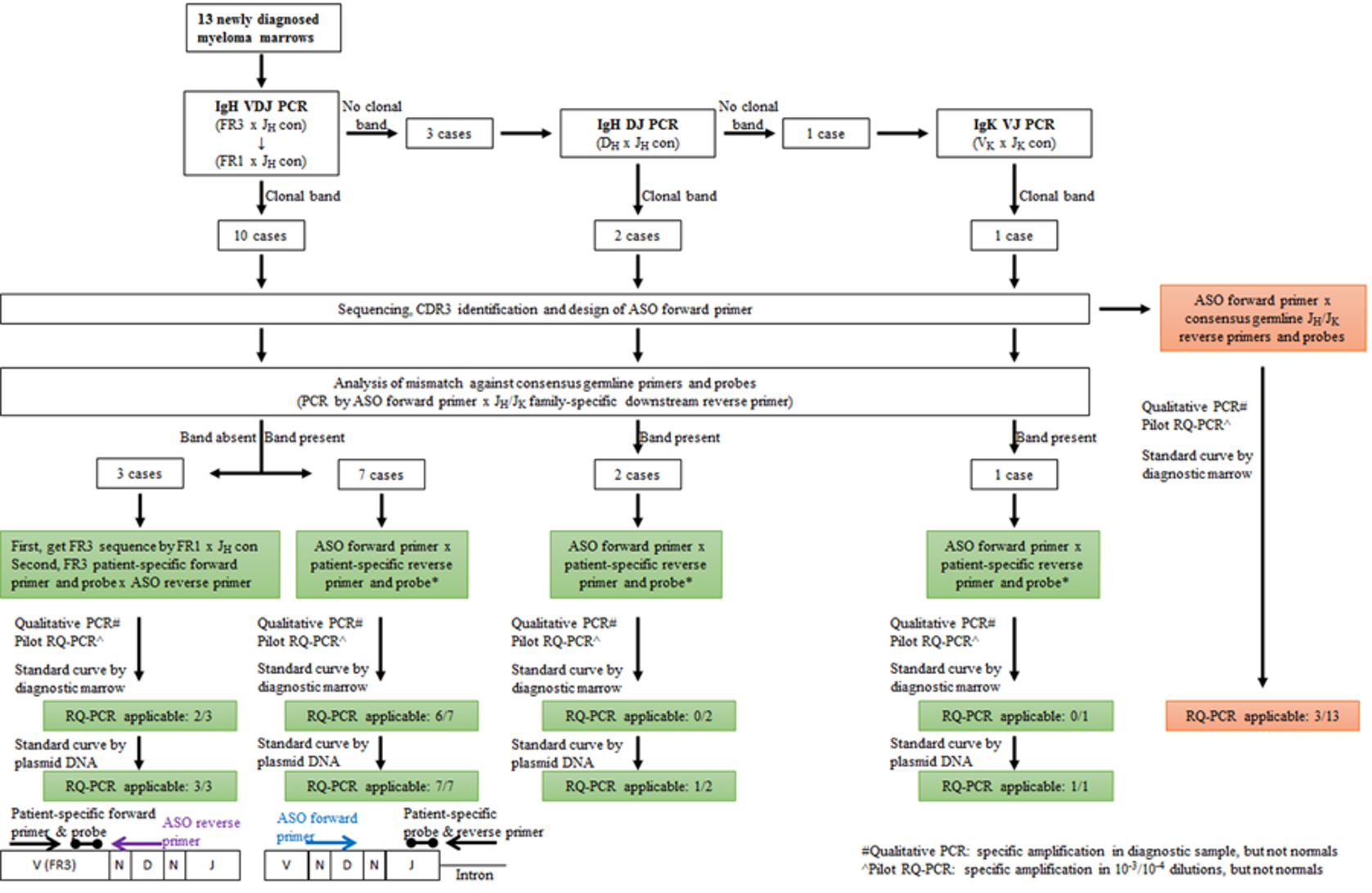


**Fig. S1** The schematic diagram for the staged approach of ASO-RQPCR in this study. * Patient-specific primers/probes were not designed for four cases [IgH VDJ (n=1), IgH DJ (n=2), IgK VJ (n=1)]: three without mismatch, hence consensus germline equals to patient-specific; one (M2) lacking a valid ASO primer due to absence of N region in the D(N)J of CDR3.

**
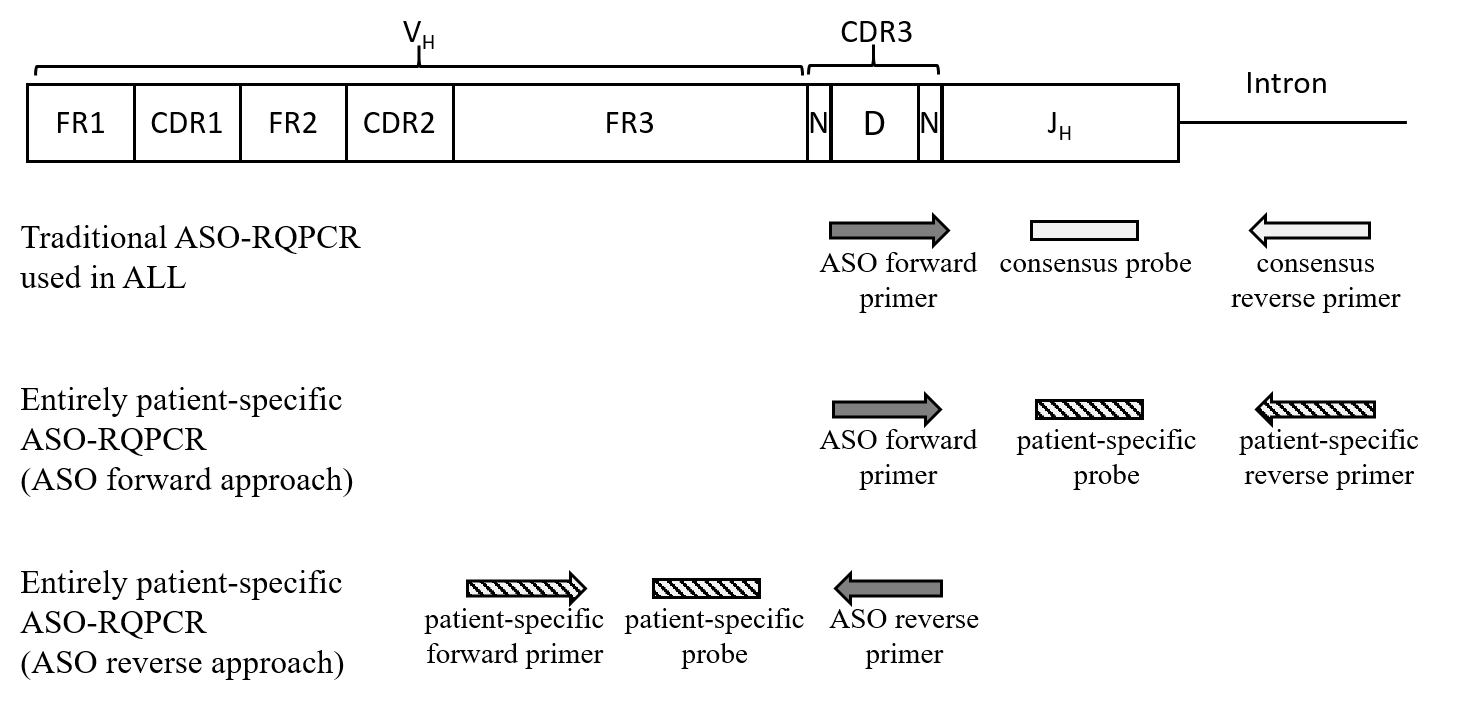
**

**Fig. S2** Principles of ASO-RQPCR used in this study. Locations of ASO primers, consensus and patient-specific primers/probes are shown. For ASO forward approach, probes and reverse primers with entirely patient-specific sequences are located similarly at J_H_ exon and intron respectively as the traditional ASO-RQPCR used in ALL [[1](#_ENREF_1)]. For ASO reverse approach, forward primers and probes with entirely patient-specific sequences are both located at V_H_ FR3 region.

**
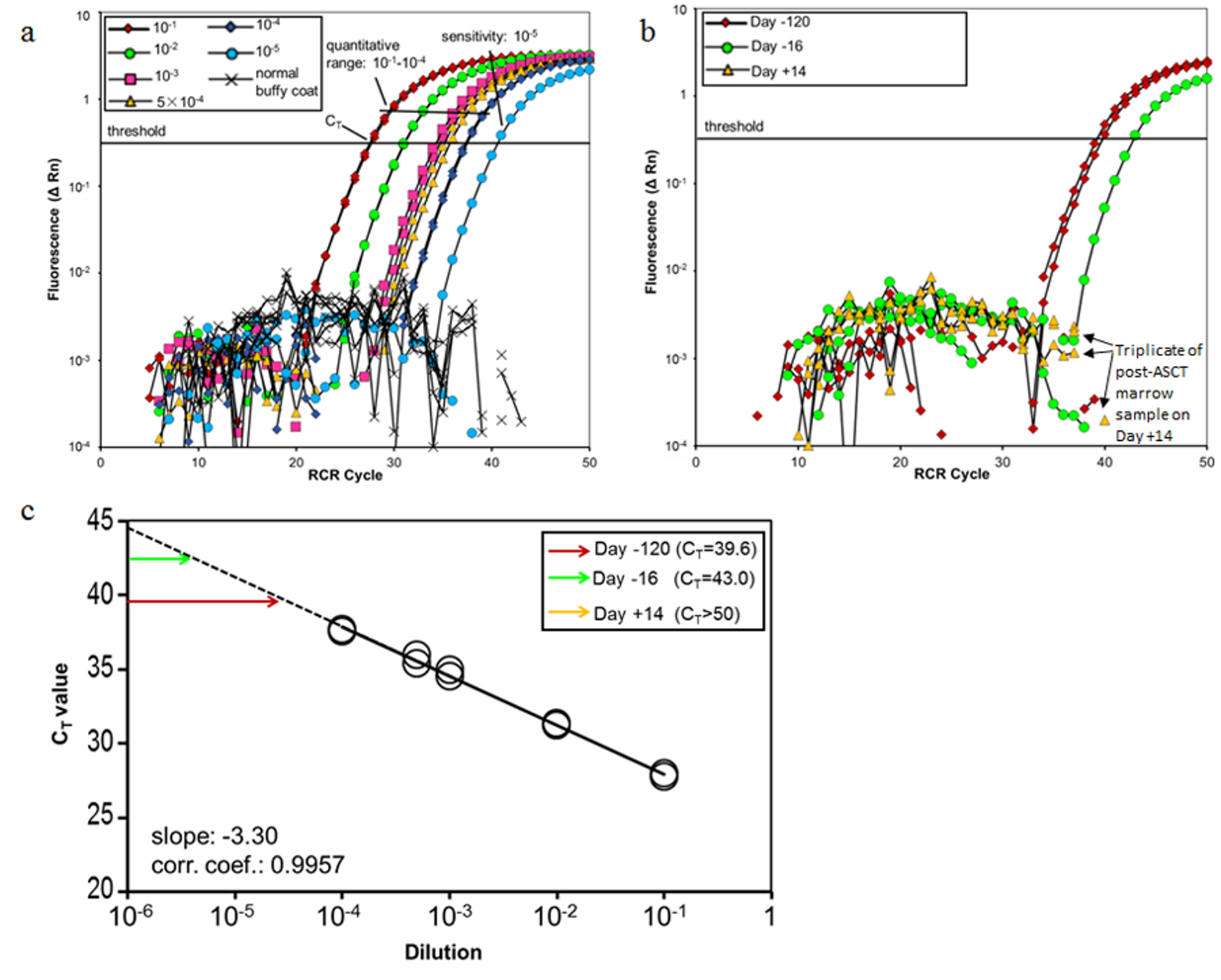
**

**Fig. S3** MRD detection in the follow-up samples of patient Dx53 by patient-specific ASO-RQPCR. (a) ASO-RQPCR showed specific amplification in serial diluted diagnostic DNA but not normal buffy coat controls (C_T_=50). According to the EuroMRD guideline, the ‘quantitative range’ herein was 10^-4^ and the ‘sensitivity’ was 10^-5^ (C_T_ value: 40.8). (b) Amplification plot of the three follow-up marrow samples showed specific amplification in the two pre-ASCT samples on Day -120 (red diamond lines) and Day -16 (green circle lines), but not post-ASCT on Day +14 (yellow triangle lines). c) Standard curve constructed from the C_T_ values of diagnostic DNA dilutions within the quantitative range (10^-1^-10^-4^) showed a slope of -3.30 and a correlation coefficient of 0.9957, hence qualified for quantification of MRD in the follow-up marrow samples. C_T_ values of the pre-ASCT follow-up marrow on Day -120 were 39.4 & 39.9 in two of the triplicate, and another pre-ASCT marrow sample on Day -16 was 43 in one of the triplicate. Therefore, both samples were MRD positive but non-quantifiable. Finally, C_T_ value of the post-ASCT marrow sample (Day +14) was >50, indicating absence of MRD.

**Table S2** Clonality detection, gene rearrangements and ASO primers used for RQ-PCR

| Cases | Clonality detection | Gene rearrangement^a^ | ASO primer sequence (5' -> 3')^b^ |
| --- | --- | --- | --- |
| Dx53 | IgH VDJ VH3-FR1 | V_H_3.11(1)-15-(5)D2.21(12)-8-(10)J_H_6 | GTCCAGCGAGAAACGGATTG |
| M3 | IgH VDJ VH3-FR1 | V_H_3.30(2)-7-(2)D3.3(15)-11-(8)J_H_4 | CTGTGCGAAAAACCGATTGC |
| M8 | IgH VDJ FR3 | V_H_1.18(4)-9-(1)D3.10(10)-20-(3)J_H_4^c^ | GAAGCGGTTCTCCCTTGGGT |
| M4 | IgH VDJ VH1-FR1 | V_H_1.24(2)-(7)D1.1(1)-12-(17)J_H_4 | GCACTGGAGCGAATGGTGG |
| Dx31 | IgH VDJ VH4-FR1 | V_H_4.61(4)-8-4-D5.18(7)-11-(6)J_H_4 | ATACAACTACGCTGCGATCCTTG |
| M5 | IgH VDJ FR3 | V_H_1.18(2)-4-(6)D2.2(12)-20-(8)J_H_3^c^ | TTATAGTATTACCTAGAGACCCGTTGGA |
| M10 | IgH VDJ VH1-FR1 | V_H_1.68(6)-4-(5)D6.13(2)-11-(14)J_H_6 | CAGCACCTGGTGTGGGTAGTAGTAG |
| Dx49 | IgH VDJ VH4-FR1 and FR3 | V_H_4.39 (1)-(7)D5.12(5)-19-(12)J_H_6 | CATACCGGAGTGATAGTAATTATATGCG^d^ |
| M1 | IgH VDJ VH3-FR1 | V_H_3.30(0)-4-(8)D5.12(9)-4-(6)J_H_4 | TAAGTCAAGGGCGCGACTC^d^ |
| M6 | IgH VDJ VH4-FR1 | V_H_4.4(2)-13-(7)D6.6(0)-3-(1)J_H_3 | AAAGTATCGAGGGGCGAACT^d^ |
| M2 | IgH DJ DH6 | D6.13(2)-0-(9)J_H_4b | GCAGCAGCTGGTACTACTGGG |
| M7 | IgH DJ DH1 | D1.26(2)-11-(5)J_H_5b | GGGAGCTACTTGTTGAGGGGATG |
| M9 | IgK VJ | V_K_1.39(1)-2-(8)J_K_1 | AGTTACAGTACCCCTCAGTCGG |

1. Numbers in parentheses show the deletion of nucleotides. Numbers between dashes indicate the inserted N region. The underlined nucleotides were included for the design of ASO primers.
2. The underlined indicates the inserted N region of the CDR3 region.
3. Due to the clonality detected by FR3 PCR, VH gene is not credible.
4. For the three cases, ASO reverse primers were designed. For other 10 cases, ASO forward primers were designed.

**Table S3** V_H_, D_H_ and J_H_ family usage in cases with IgH VDJ rearrangements

| V_H_ family (n = 8)^a^ | |  | D_H_ family (n = 10) | |  | J_H_ family (n = 10) | |
| --- | --- | --- | --- | --- | --- | --- | --- |
| family | % (n) |  | family | % (n) |  | family | % (n) |
| 1 | 25% (2) |  | 1 | 10% (1) |  | 1 | --- |
| 2 | --- |  | 2 | 20% (2) |  | 2 | --- |
| 3 | 37.5% (3) |  | 3 | 20% (2) |  | 3 | 20% (2) |
| 4 | 37.5% (3) |  | 4 | --- |  | 4 | 50% (5) |
| 5 | --- |  | 5 | 30% (3) |  | 5 | --- |
| 6 | --- |  | 6 | 20% (2) |  | 6 | 30% (3) |
| 7 | --- |  | 7 | --- |  |  |  |

1. Two cases were excluded, in which VH gene was not identified as clonality was defined detected by FR3 PCR.

**Table S4** Patient-specific primers and Taqman probes used for RQ-PCR and downstream primers for detecting mismatch

| Primer or probe name | | sequence (5' -> 3') | For cases |
| --- | --- | --- | --- |
| Patient-specific primers | |  |  |
|  | Dx53 pt RP | CAGAGAGGAAGGGCCCTAGAGT | Dx53 |
|  | M3 pt RP | ATCAGAGAAAATATCACAGAGAGGTTGT | M3 |
|  | M8 pt RP | AGAGCTAAAGCAGGAGAGAGGTTGT | M8 |
|  | M4 pt RP | AGGATTTAAGTAGGGGAGACGTTGT | M4 |
|  | Dx31 pt RP | CGTAGACCCAAAGTAGGAGAGACATTA | Dx31 |
|  | Dx49 FR3FP | AGAGTCGGGTCACCATGTCAG | Dx49 |
|  | M1 FR3FP2 | CAGACACAACCCTGAATACATTGAA | M1 |
|  | M6 FR3FP2 | CTCAAGAGTCGCGTCACCATAT | M6 |
| Patient-specific probes | |  |  |
|  | T-IGJH6 | AAGGGACCACGGTCACCGTCTCCT | Dx53 |
|  | T-JH4Dx31 | TCCTGGTCACCGTCTCCTCAGGTG | Dx31 |
|  | T-JH3M5 | CCAGGGACAATGGTCACCGTCTCTTC | M5 |
|  | T-Dx49 | CCATCTTCAGGGAGAACTGATTCTTGGACG | Dx49 |
|  | T-M1 | AATAAACACCCCGGTCCTCTGTTCTCAGA | M1 |
|  | T-M6 | TCACCTTCAGGGAGAACTGGTTCTTGGAC | M6 |
| JH or JK downstream primers | |  |  |
|  | JH3 RP dst | GCTCCAGGACAGAGGACGCT | M5, M6 |
|  | JH4 RP dst | TGCTCCGGGGCTCTCTTG | M3, M8, Dx31, M1, M2 |
|  | JH5 RP dst | CTTTCTTTCCTGACCTCCAAAATG | M4, M7 |
|  | JH6 RP dst | AAGGAAACCCCACAGGCAGTA | Dx53, M10, Dx49 |
|  | JK1 RP dst | TGAAGGATATCAGAGGCTGATTGCAGA | M9 |

***References***

1. Verhagen OJ, Willemse MJ, Breunis WB, Wijkhuijs AJ, Jacobs DC, Joosten SA, et al. Application of germline IGH probes in real-time quantitative PCR for the detection of minimal residual disease in acute lymphoblastic leukemia. Leukemia. 2000; 14:1426-35.
